# The Right Heart of Elite Male and Female Cyclists: A Comparative Study of Cycling Disciplines

**DOI:** 10.3390/jcdd13070303

**Published:** 2026-07-01

**Authors:** Max Knights, Aneil Malhotra, Robert Cooper, Shaun Robinson, Tristan Ramcharan, Joseph Maxwell, Jersusalem Fekadu, Camille S. L. Galloway, Florence Place, Keith George, Nigel Jones, David Oxborough

**Affiliations:** 1 Salford Royal, Northern Care Alliance NHS Foundation Trust, Salford M6 8HD, UK; max.knights@nca.nhs.uk; 2 Institute of Sport, Manchester Metropolitan University, Manchester M1 7EL, UK; aneilmalhotra@gmail.com; 3 Research Institute for Sport and Exercise Sciences, Liverpool John Moores University, Liverpool L3 3AF, UK; rob.cooper@lhch.nhs.uk (R.C.); shaunrobinson@nhs.net (S.R.); j.d.maxwell@ljmu.ac.uk (J.M.); f.place@2024.ljmu.ac.uk (F.P.); k.george@ljmu.ac.uk (K.G.); 4 Liverpool Heart and Chest Hospital NHS Foundation Trust, Liverpool L14 3PE, UK; 5 Imperial College Healthcare NHS Foundation Trust, London W2 1NY, UK; 6 Heart Unit, Birmingham Women’s and Children’s NHS Foundation Trust, Birmingham B4 6NH, UK; tristanramcharan@doctors.org.uk; 7 Guy’s and St Thomas’ NHS Foundation Trust, London SE4 7EH, UK; 1f.jerry@gmail.com; 8 School of Health and Exercise Sciences, University of British Columbia Okanagan, Kelowna, BC V1V 1V7, Canada; camille.sophie.galloway@gmail.com; 9 Medical Department, British Cycling, Manchester M11 4DQ, UK; nigeljones@britishcycling.org.uk

**Keywords:** athletes heart, right heart, echocardiography, strain, elite cyclists

## Abstract

Background: Elite cycling can be dichotomised into road (endurance cycling) and track (sprint cycling) events that are associated with different training components and athlete physiology. Evidence of disproportionate right ventricular (RV) remodelling in endurance athletes places importance on defining physiological and athletic phenotypes. Therefore, the elite cyclist serves as an ideal model to study training-specific adaptations in the right heart. Methods: One hundred and eighty-six (110 males and 76 females) elite international-level cyclists (mean age 23 ± 5 years) were grouped by discipline (endurance cycling and sprint cycling) and sex (male cyclists and female cyclists). RV and right atrial (RA) structure and function was assessed with 2D, tissue Doppler, and strain echocardiography. Structural data were scaled allometrically to body size. Group comparisons were made with a two-way ANOVA. Results: Significantly larger absolute and scaled RV and RA structural values were seen in male and endurance cyclists (RV end diastolic area index: male endurance 14 ± 2 cm^2^/m^2^, male sprint 13 ± 2 cm^2^/m^2^) than female and sprint cyclists (female endurance 13 ± 2 cm^2^/m^2^, female sprint 11 ± 2 cm^2^/m^2^), respectively. Endurance training exposure was significantly correlated to structural parameters (RA area index: *r* = 0.53; *p* < 0.001). Sex and discipline showed significant impact on resting RV function with reduced RV strain values in male and endurance cyclists (RV basal strain: male endurance −17.5 ± 4.0%, male sprint −19.4 ± 4.6%) compared to female and sprint cyclists (female endurance −19.9 ± 3.9%, female sprint −22.1 ± 5.1%), respectively. There were no significant correlations between training exposure and resting RV function. Conclusions: Athlete sex and cycling discipline exert significant effects on RV and RA structure and function in elite cyclists. The strong correlations observed between endurance training exposure and right heart structure emphasise the importance of consideration of sporting discipline in elite athletes.

## 1. Introduction

The ‘athlete’s heart’ reflects the morphological, functional, and electrical cardiac adaptations associated with structured exercise training [1,2]. Profound physiological dilatation of all cardiac chambers can occur in endurance-trained athletes [3,4,5], whereas similar adaptation has not been demonstrated in pure strength-trained athletes [6]. Elite-level cycling comprises both endurance and sprint cycling, with differing training loads. Endurance cycling requires the athlete to undertake both isometric and isotonic exercise components for prolonged periods of time [7], and studies have highlighted marked structural cardiac adaptation in this population [8,9]. However, elite sprint cyclists train at higher intensities for shorter periods of time, and therefore, this population can serve as a controlled comparison of training exposure, within the same sport, upon cardiac structural and functional adaptation.

Intriguingly, right heart data in athletes suggests disproportionate right ventricular (RV) remodelling in endurance athletes [10,11] potentially related to higher relative RV wall stress during prolonged aerobic exercise [12]. There is also evidence demonstrating predominant RV-based exercise-induced cardiac fatigue and cardiomyopathy in endurance athletes [10,13]. Individual cardiac adaptation to training has been strongly associated with overall training volume and cardiorespiratory fitness [7,14,15,16]. These data, however, are limited by the comparison of athletes from different sports, where there is the potential for a confounding impact of training exposure, exercise posture, and their associated active muscle groups [17,18]. The right atrium (RA) also reflects the impact of volume and pressure stimuli during and in response to chronic exercise exposure [19,20]. There are data demonstrating the importance of RA structure and function on RV physiology [21,22], and hence the nature of this coupling may provide additional valuable information on the impact of a variable training load on the right heart phenotype in an elite cycling cohort.

Athletic heart research is predominantly focused on male athletes, although those studies that have compared sexes [23] highlight that female athletes typically present with lesser absolute but similar indexed structural parameters compared to male athletes, alongside some evidence of augmented functional parameters [5,10,23,24,25]. The comparison of RV and RA structure and function in elite-level male and female cyclists competing in both endurance and sprint disciplines has not been previously documented.

In view of this, the aim of this study was to compare RV and RA structural and functional phenotypes of elite male and female endurance and sprint cyclists.

## 2. Materials and Methods

One hundred and ninety-two elite international-level cyclists were assessed as part of their national cycling team’s pre-participation screening programme. All cyclists conformed to the World Anti Doping Agency (WADA) regulations. They were included in the study if they had no history of cardiovascular disease, were not taking any cardiovascular medications, had a normal 12-lead electrocardiogram (ECG), and had no echocardiographic evidence of cardiac disease. Subsequently, 186 cyclists consented to be included in the final data analysis, including 110 males (23 ± 6 years) and 76 females (23 ± 5 years). Ethics approval was obtained from the National Research Ethics Service at London–West London & GTAC Research Ethics Committee (IRAS 169429), with all cyclists providing written informed consent.

All data were acquired at rest in a single session with cyclists refraining from any strenuous physical activity for at least 6 h prior to the examination or consuming caffeine or alcohol within 24 h of examination. Cyclists initially completed a health and training questionnaire and were then allocated into either endurance (track endurance, road, mountain bike, cyclocross, tandem pilot) (*n* = 124) or sprint (track sprint, tandem, kilo team, BMX) (*n* = 62) groups and further sub-grouped by sex (Male Endurance [ME] *n* = 71, Male Sprint [MS] *n* = 39, Female Endurance [FE] *n* = 53, Female Sprint [FS] *n* = 23). The cohort was predominantly of white ethnicity (96.2%). Metabolic Equivalents (METS) data were obtained for each aspect of training in accordance with the 2024 Adult Compendium of Physical Activities [26]. Cyclists’ training included: endurance cyclists (endurance training 66.7%, 12.8 METS; sprint training 22.2%, 16.8 METS; strength training 11.1%, 7 METS) and sprint cyclists (endurance training 26.1%, 12.8 METS; sprint training 43.5%, 16.8 METS; strength training 30.4%, 7 METS). These values were multiplied by cyclists’ training hours per week to obtain total METS and MET hours per week, endurance METS and MET hours per week, sprint METS and MET hours per week, and strength METS and MET hours per week.

Anthropometric assessment included height (Seca 217, Hannover, Germany) and body mass (Seca supra 719, Hannover, Germany). Body surface area (BSA) was calculated using the Mosteller equation [27]. Resting systemic blood pressure was assessed with an automated sphygmomanometer (Dinamap 300, GE Medical Systems, Chicago, IL, USA). A resting 12-lead ECG was also performed.

### 2.1. Echocardiography

A full transthoracic echocardiogram was completed by a British Society of Echocardiography (BSE) accredited sonographer, adhering to BSE national guidelines [28,29,30], and reviewed during analysis by a second BSE-accredited sonographer. Echocardiographic images were acquired with a commercially available ultrasound system (Vivid IQ or Vivid E95, GE Medical, Horten, Norway) and a 1.5–4 MHz phased array transducer. Data was stored in raw digital imaging and communications in medicine (DICOM) format and transferred to an offline workstation (EchoPAC, Version 204, GE Healthcare, Horten, Norway). All structural values were scaled based on the principles of geometric similarity [11] scaled to powers of BSA, with exponents of ^0.5^ for linear dimensions, ^1.0^ for areas and ^1.5^ for volumes [31].

RV dimensions were measured from the RV-focused apical 4-chamber view (A4C) in diastole at the RV basal-level diameter (RVD_1_), mid-level diameter (RVD_2_), and length from base of tricuspid annulus to apex (RVD_3_). RV and left ventricle (LV) end diastolic diameters were also measured in standard A4C to establish the RV:LV ratio. The RV outflow tract (RVOT) was measured from the parasternal long and short axis windows in diastole at the RV anterior wall to the interventricular septum/aortic wall (RVOT_plax_), from the anterior aortic wall directly to the RV anterior wall (RVOT_1_), and proximal to the pulmonary valve (RVOT_2_) locations. RVOT_1_:RVD_1_ ratio was calculated to establish relative outflow to inflow dimensions. RV end diastolic area (RVEDA) and end systolic area (RVESA) were measured from the RV-focused A4C view, from which the RV fractional area change (RVFAC) was calculated. Tricuspid annular plane systolic excursion (TAPSE) was measured using M-Mode in the A4C view, and peak RV lateral systolic (RV S’), early diastolic (RV E’) and late diastolic (RV A’) myocardial velocities were measured using tissue Doppler imaging. LV cavity dimensions were measured in the parasternal short axis view perpendicular (D1) and parallel (D2) to the septum in diastole and systole to calculate the eccentricity index (D2/D1) in systole (EIs) and diastole (EId).

RA area and volumes (end ventricular systole [RAESV], pre-atrial contraction, and end ventricular diastole [RAEDV]) were measured in the A4C view. RA volumes at these specific points in the cardiac cycle enabled calculation of reservoir (RARV; RAESV–RAEDV), conduit (RACV; RAESV–pre-atrial contraction), and booster volumes (RABV; pre-atrial contraction–RAEDV). Inferior vena cava (IVC) diameter was measured in the subcostal view using the maximum IVC dimension.

Myocardial strain (ε) indices were analysed by speckle tracking echocardiography (STE) using a semiautomated 2-D offline software package (EchoPAC, Version 204, GE Healthcare, Horten, Norway). Assessment of global longitudinal ε for the RV free wall (RVFW) and regional longitudinal ε was calculated using a frame rate of between 40 and 90 frames per second in the modified RV-focused A4C. The region of interest was positioned around the RVFW and septum to maximise tracking, with only RVFW ε, RV basal ε (RVBS), RV mid ε (RVMS), and RV apical ε (RVAS) segments being reported [32]. A base-to-apex (B-A) gradient was calculated by assessing percentage differences between basal and apical ε. RA ε was assessed by peak reservoir atrial longitudinal ε (RARS), atrial conduit ε (RACS), and atrial booster ε (RABS). Previous work from our laboratory has reported excellent STE RV ε intra-observer reproducibility with an intra-class correlation coefficient (ICC) of 0.843 and a coefficient of variation (CoV) of 7% [33,34].

### 2.2. Statistical Analysis

Continuous data are presented separately for sex and discipline as mean ± standard deviation (SD). Data was analysed for normal distribution using the Kolmogorov–Smirnov test followed by a two-way (sex and discipline) between-group ANOVA. Tukey’s post hoc correction was applied where appropriate to adjust for multiple comparisons. A *p*-value < 0.05 was considered significant. Pearson’s correlation tests were used to determine relationships between training volume data and right heart variables. The smallest worthwhile correlation coefficient effect was defined as *r* = 0.30, a moderate effect size in Cohen’s terms [35]. Statistical analysis was performed using GraphPad Prism (Version 10.0.0 for Mac, GraphPad Software, Boston, MA, USA) software.

## 3. Results

### 3.1. Demographics and Training Volume Characteristics

Demographics, anthropometrics, and training volumes are detailed in Table 1. Sprint cyclists were significantly heavier and had a larger BSA (MS, 2.01 ± 0.15 m^2^; FS, 1.77 ± 0.09 m^2^) than endurance cyclists (ME, 1.91 ± 0.13 m^2^; FE, 1.67 ± 0.10 m^2^). Endurance cyclists had significantly lower resting heart rates (HR) (ME, 54 ± 10 bpm; FE, 58 ± 11 bpm) compared to sprint cyclists (MS, 60 ± 9 bpm; FS, 62 ± 9 bpm; *p* = 0.004). Systolic blood pressure (SBP) was significantly higher in male cyclists (ME, 124 ± 12 mmHg; MS, 128 ± 12 mmHg) than female cyclists (FE, 120 ± 13 mmHg; FS, 115 ± 14 mmHg; *p* < 0.001). There were significant differences observed between disciplines for all training volume parameters, other than training duration (years). Total METS per week were significantly greater in endurance cyclists (ME, 240 ± 50 METS; FE, 221 ± 42 METS) than sprint cyclists (MS, 199 ± 71 METS; FS, 206 ± 62 METS; *p* = 0.001).

### 3.2. Right Ventricular Structure

**Figure 1:**
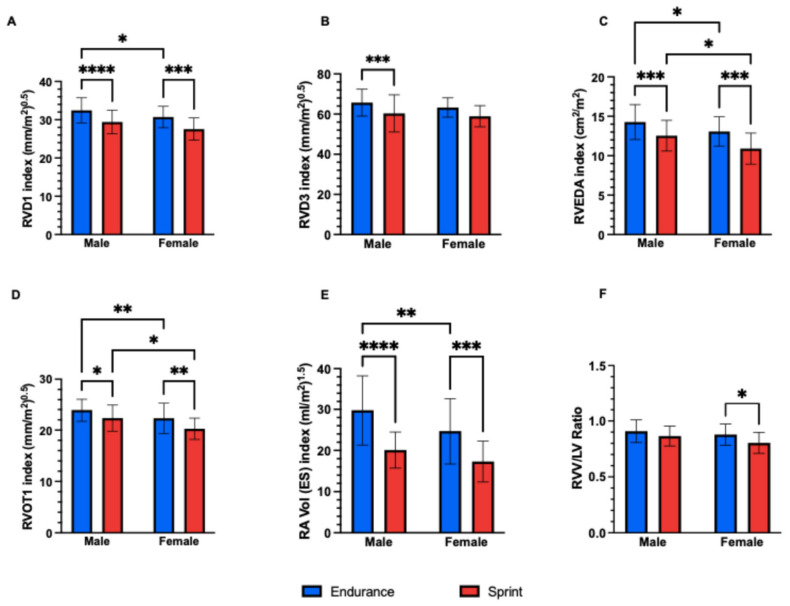
Six-panel plot of RV and RA structural dimensions in male and female sprint and endurance cyclists. (**A**) RVD1 index; (**B**) RVD3 index; (**C**) RVEDA index; (**D**) RVOT1 index; (**E**) RA end systolic volume; (**F**) RV/LV ratio. Bars represent mean ± SD. * = *p* < 0.05, ** = *p* < 0.01, *** = *p* < 0.001, **** = *p* < 0.0001.

RV structural parameters are displayed in Table 2. There were significant main effects (*p* < 0.020) of discipline for RVOT_plax_ index, RVOT_1_ index, RVOT_2_ index, RVD_1_ index, RVD_2_ index, RVD_3_ index, RVEDA index, RVESA index and RV:LV ratio, with larger RV structural values observed in endurance cyclists than sprint cyclists (RVEDA index mean difference 1.96 cm^2^/m^2^, 95% CI 1.28 to 2.63, *p* < 0.0001), regardless of sex (Figure 1). We observed significant main effects (*p* < 0.002) of sex for RVOT_1_ index, RVOT_2_ index, RVD_1_ index, RVD_2_ index, RVEDA index, RVESA index, RV:LV ratio and EIs, with larger RV structural values observed in male cyclists compared to female cyclists (RVD_1_ index mean difference 1.79 mm/m^2(0.5)^, 95% CI 0.82 to 2.76, *p* < 0.0001; Figure 1). There were no significant differences between groups for RVOT1:RVD1 ratio or EId. No significant interaction terms were observed.

**Figure 2:**
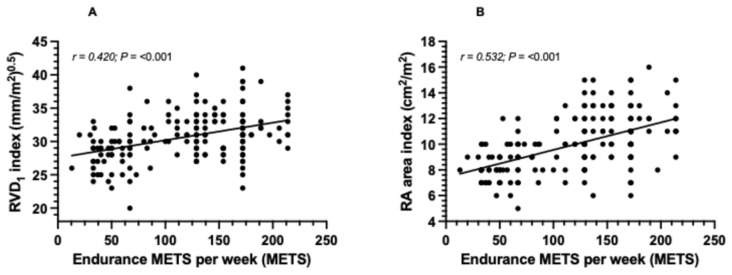
Pearson’s correlation coefficient of endurance METS per week and (**A**) RVD_1_ index; (**B**) RA area index.

Endurance METS per week was significantly (*p* < 0.001) correlated to RVD_1_ index (*r* = 0.42) (Figure 2), RVEDA index (*r* = 0.38), RVD_3_ index (*r* = 0.37), RVOT_1_ index (*r* = 0.35), RVOT_plax_ index (*r* = 0.35), RVESA index (*r* = 0.35), and RVOT_2_ index (*r* = 0.35).

### 3.3. Right Ventricular Function

**Figure 3:**
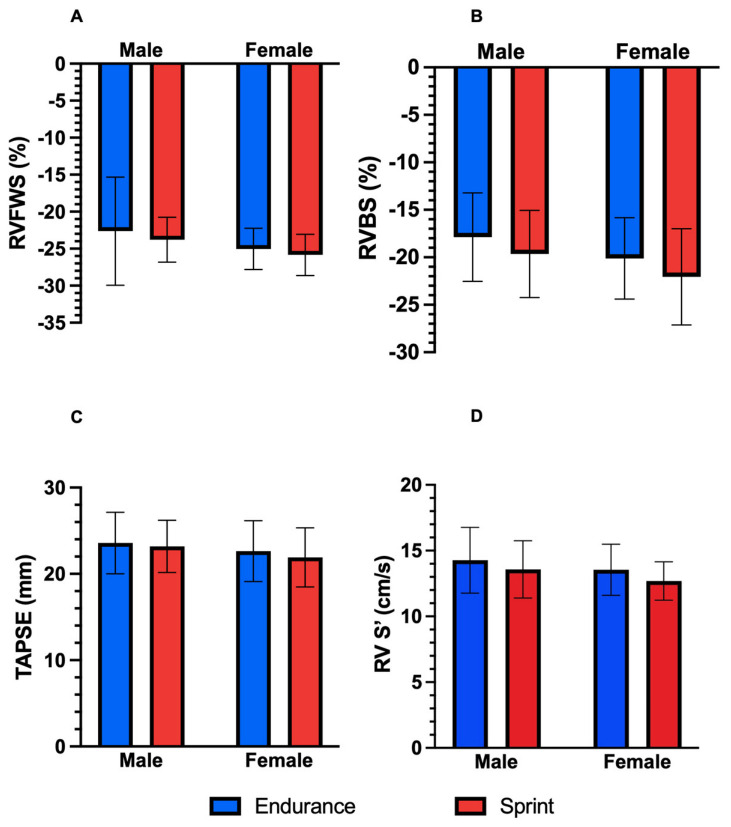
Four-panel plot of RV functional parameters in male and female sprint and endurance cyclists. (**A**) RVFWS; (**B**) RVBS; (**C**) TAPSE; (**D**) RV S′. Bars represent mean ± SD. The ANOVA indicates a significant main effect (*p* < 0.05) despite a non-significant pairwise interaction.

RV functional parameters are displayed in Table 2. Discipline showed a significant main effect on RV S′ (*p* = 0.028), RVBS (*p* = 0.024), and B-A gradient (*p* = 0.020), with greater RVBS and B-A gradient values in sprint cyclists (RVBS mean difference 1.85%, 95% CI 3.44 to 0.25, *p* = 0.024), whereas RV S′ data were greater in endurance cyclists. There were significant differences between sexes for TAPSE (*p* = 0.047), RV S′ (*p* = 0.022), RVFWS (*p* = 0.012), RVBS (*p* = 0.005), and RVAS (*p* = 0.009), with higher RVFWS, RVBS, and RVAS values and lower TAPSE and RV S′ values in female cyclists compared to male cyclists (RVFWS mean difference 2.23%, 95% CI 0.49 to 3.97, *p* = 0.01; Figure 3). Weak but significant (*p* < 0.0005) correlations of RVD_3_ and RVD_3_ index to TAPSE (RVD_3_: *r =* 0.29; RVD_3_ index: *r =* 0.25) and RV S′ (RVD_3_: *r =* 0.28; RVD_3_ index: *r =* 0.28) were observed. No significant differences between groups were observed for RV E′, A′, and RV E′/A′ ratio. No significant interaction terms were observed.

Endurance METS per week were weakly but significantly inversely correlated to RVBS (r = −0.16; *p* = 0.047) and B-A gradient (r = −0.18; *p* = 0.032). No other significant correlations between training volumes and RV function were observed.

### 3.4. Right Atrial Structure and Function

RA structural and functional parameters are displayed in Table 3. There were significant main effects (*p* < 0.001) of discipline on RA area index, RAESV index, RARV index, RACV index, RABV index, and IVC diameter, with larger RA structural parameters in endurance cyclists than sprint cyclists (RA area index mean difference 2.49 cm^2^/m^2^, 95% CI 1.93 to 3.06, *p* < 0.0001; Figure 1). We identified that sex showed significant main effects (*p* < 0.008) for RA area index, RAESV index, RARV index, RABV index, and IVC diameter, with larger mean data observed in male cyclists compared to female cyclists (RAESV index mean difference 8.54 mL/m^2(1.5)^, 95% CI 6.20 to 10.87, *p* = 0.001; Figure 1). No significant differences were observed between groups for RA ε indices. No significant interaction terms were observed.

Endurance METS per week was significantly correlated (*p* < 0.001) to RA area index (*r* = 0.53), RAESV index (*r* = 0.49), IVC diameter (*r* = 0.38), and RARV index (*r* = 0.32) (Figure 2). No other significant correlations between training volumes and RA function were observed.

## 4. Discussion

The main findings from this study were: (i) absolute and body-size index values for RV and RA structural indexes were greater in male cyclists and endurance cyclists compared to female cyclists, and sprint cyclists (ii) sex and discipline showed significant impact on measures of resting RV function and (iii) there was a significant and positive correlation between greater amount of endurance training exposure and larger RV and RA structural parameters.

### 4.1. Right Ventricular Structure

It has been previously documented that endurance athletes have globally augmented RV dimensions, whereas resistance athletes exhibit similar RV structural dimensions to sedentary controls [6,11,36,37,38]. RV anatomy and function are complex [39], mainly composed of superficial muscle layers and thin walls [40] being sensitive to preload, giving rise to physiological RV remodelling after exposure to high wall stress and cardiac output (CO) during intense exercise [41,42]. The RV in endurance cyclists is likely to be exposed to increased pulmonary artery pressures (PAP) and wall stress for sustained periods of time. During aerobic exercise, the pulmonary circulation has a limited ability to decrease vascular resistance compared to the systemic circulation, demonstrated by a linear relationship between CO and PAP [43,44]. Increases in left atrial pressure during exercise subsequently raise pulmonary capillary wedge pressure and RV systolic pressures [45] and therefore, during aerobic exercise, the RV is exposed to greater load and relative wall stress over longer periods of time due to disproportionate afterload increases. The short cyclical increases in CO seen in sprint cycling may therefore be insufficient stimulus to cause the analogous adaptation seen in endurance cyclists. However, no direct physiological measurements of PAP were collected during this study to explicitly support this theory.

Interestingly, our data contradicts previous research [10], which denoted that RV:LV ratio does not differ by sporting discipline, yet may be augmented when compared to non-athletic controls. Findings from our study showed that endurance cyclists showed a significantly greater RV:LV ratio than sprint cyclists, which is expected to be reflective of enhanced physiological remodelling seen in endurance cyclists than in sprint cyclists. Though we observed increased RV structural adaptations, the RVOT_1_:RVD_1_ ratio was not raised and showed no significant differences between groups, suggesting proportionate remodelling within the RV chamber in elite cyclists. It is important to acknowledge that from the data presented within this study alone, we cannot provide conclusive differentiation from other mechanisms of cardiac remodelling.

Sex differences in the athlete’s heart are often overlooked, particularly for the right heart. Our data suggest that sex has a significant independent influence on RV structure, with male cyclists presenting with larger absolute and indexed structural RV dimensions than female cyclists, regardless of discipline. These findings contrast with previous research in a large cohort of Olympic athletes [10] where authors found absolute RV dimensions were greater in male athletes than female athletes, but when indexed to linear dimensions, female athletes showed larger dimensions than male athletes. Indices from our study were indexed to BSA allometrically, and therefore, we can rationalise that using a more precise scaling methodology provides more accurate findings.

Research suggests several potential hypotheses as to why we observed pronounced differences in RV structure between sexes. Firstly, androgenic receptors are present in myocardial tissues, allowing circulating androgens to play a role in modulating the cardiac phenotype [46]. These receptors modulate the hypertrophic response in cardiac myocytes, with research evidencing increased levels of hypertrophy in males compared to age-matched females [47]. However, in the absence of hormonal measurements and analysis, mechanistic speculation cannot be inferred from the data within this study. Additionally, it could be speculated that these differences may, in part, be due to the vasodilator effect of oestrogens on pulmonary vasculature and therefore act as a protective mechanism in females [23,48,49]. Secondary influences such as training volume, exercise intensity, muscle mass, and blood volume expansion should also be considered within this context [25]. There were no significant differences observed in our cyclists’ training volume between sexes, but individual exercise intensity was not accounted for.

To the best of our knowledge, this study is the first to assess the impact of discipline-specific training on male and female elite cyclists. We observed that the cyclists completing greater amounts of endurance METS per week demonstrated greater RV and RA structural dimensions. The equivalent magnitude of structural adaptations was not seen for sprint or strength METS per week, emphasising the importance of consideration for sporting discipline and training volume upon assessment of the athlete’s heart. This finding also supports previous theories that a dose–response relationship exists for endurance training protocols [50]. However, it must be noted that although sprint cycling training adaptations were not to the same magnitude as endurance cycling, the group still exhibits increased structural dimensions compared to non-athletic cut-offs, likely due to having a proportion of their training dedicated to endurance, albeit at a lower volume and intensity than pure endurance cyclists.

### 4.2. Right Ventricular Function

Research on RV function in athletic populations is equivocal, with some studies showing reduced RV function [13,51,52] in endurance athletes, whereas others identify no evidence of reduced indices [11,53,54]. Our study observed enhanced RVBS and subsequently lower B-A gradient in sprint cyclists compared to endurance cyclists, and this finding of reduced RVBS at rest is consistent with previous research [51,54,55]. Weak yet significant correlations between RV function and RV structure were observed in this cohort, and it is therefore possible that these findings may reflect the reduced contractility requirement due to greater RV dimensions, providing adequate SV and CO for less myocardial deformation. However, in the presence of normal wall thickness, the ‘La Place Law’ theorises this will manifest at the expense of increased wall stress, a risk factor for myocardial damage [56]. This finding highlights a previously observed relationship between structure and function [51,52], and further research has demonstrated that exercise stress testing induces normalisation of function in athletes [52]. Additionally, the preserved indices of RV diastolic function (RV E′, RV A′ and RV E′/A′ ratio) and no significant differences between groups are suggestive of preserved RV diastolic function in our cohort of elite cyclists.

Weak yet statistically significant inverse correlations between endurance METS per week and both RVBS and B-A gradient were identified, suggesting that the more endurance METS per week were completed, the lower basal ε values were observed. This may be explained by endurance cyclists having a greater apical dependence for RVFW ε [37] due to contractile reserve or that endurance cyclists demonstrate a loss of RV convexity subsequent to RV dilatation [57]. In support of this, we observed weak yet significant correlations of RV longitudinal diameter (RVD_3_ and RVD_3_ index) to longitudinal systolic functional parameters (TAPSE and RV S′). Previous research on the LV found long-axis myocardial tissue velocities (S′) to be proportional to LV length and heart size [58,59] and therefore these principles may explain the augmented longitudinal systolic RV indices in those cyclists with larger RV structural parameters.

It is important to note that other indices of RV function (i.e., TAPSE, RV S′ and FAC) were within normal limits. The use of conventional 2-D echocardiography may be limited, as this finding highlights the importance of ε imaging and its ability to detect discrete regional RV functional changes, magnifying the significance of including regional ε in routine athletic assessment.

### 4.3. Right Atrial Structure and Function

Few studies have explored the RA phenotype in elite athletes [5,6,16,21,25,53,60,61,62] and to the best of our knowledge, prior studies assessing the impact of training volume on RA parameters were categorised by skill and not volume [19]. Therefore, this study provides novelty to the assessment of the RA in elite athletes and the impact of training volume.

The RA is an important contributor to RV filling, particularly in conditions of greater venous return. Likewise, to the RV, RA structural dimensions were significantly greater in endurance cyclists than in sprint cyclists and in male cyclists than in female cyclists. Similar mechanisms can therefore be expected to be responsible for the augmented RA structural dimensions observed between disciplines and sexes, as previously discussed.

Our functional data is comparable with previously published literature [61], with no observed decline seen in RA functional parameters in the elite cyclist cohort. Likewise, training volume did not show a significant correlation with RA reservoir, conduit, or booster ε indices. From this finding, we may consider that the preserved RA functional mechanics may be more closely governed by RV remodelling than a dose–response relationship with training exposure. Although no significant difference between groups was observed, FE demonstrated the greatest RA ε values, which interestingly differs from RV ε findings, where FS demonstrated the greatest ε indices. We can speculate that the structurally larger RV has lower ε indices due to contractile reserve, and the structurally larger RA has enhanced ε indices due to improved compliance, caval return, and RV shortening.

### 4.4. Limitations

Most athletes examined were white (96%), and therefore, the effects of ethnicity and ethnic diversity were not assessed. However, due to the homogenous ethnic population, comparisons between discipline and sex can be made without any confounding effect from ethnicity. Additionally, previous research quantified ethnicity to not significantly (2.3% variation in RVOT size) impact RV structural and functional indices [63]. Due to the prolific influence of genetic adaptation, we cannot exclude or assess the extent of cardiac adaptation that is secondary to genetic cause and not intensive exercise.

The data were obtained at rest only, and therefore, we cannot assess the presence of physiological contractile reserve, particularly for those with reduced RVBS. Additionally, the use of 3- or 4-Dimensional echocardiography was not available for use at the time of this study, which has proven pivotal for RV (RVD_1_ and RVD_2_) and tricuspid valve measurement accuracy [64]. As a cross-sectional study, the timing of structural adaptation cannot be determined, and seasonal variation may influence cardiac evaluation. Moreover, cross-sectional studies do not allow the establishment of cause-and-effect relationships or dose–response relationships.

MET-derived estimations of training load were used in this study without the use of objective physiological parameters (i.e., maximal oxygen consumption (VO_2_max), lactate threshold) to determine exercise intensity and individual exercise intensity. We acknowledge that METS lack individualization and are a greater measure of external load (speed, distance, and power) rather than internal load (HR and VO_2_max) and, therefore, cardiovascular workload.

## 5. Conclusions

Endurance cycling training is linked to greater right heart structural remodelling than sprint training in elite cyclists. Sex and training discipline have both strong and independent influences on resting RV and RA functional adaptations in elite cyclists. Training discipline and exposure are key determinants of structural and functional changes in elite cyclists. Whilst the present study was not designed to define normative reference values, the findings are compelling for clinicians to consider athlete-specific characteristics. Therefore, these discipline- and sex-specific findings should be considered when interpreting the expected magnitude of right heart adaptation in this population and may be used to inform future athletic pre-participation screening guidance.

## Figures and Tables

**Table 1:** Demographic, anthropometric, and training volume characteristics of elite cyclists.

	Female		Male	
	Sprint	Endurance	Sprint	Endurance
Age (y)	22 ± 3	23 ± 5	23 ± 5	23 ± 6
Height (cm) *^b^*	164 ± 6	166 ± 6	179 ± 7	180 ± 7
Weight (kg) *^a^*^,*b*^	69 ± 5	61 ± 6	81 ± 9	73 ± 8
BSA (m^2^) *^a^*^,*b*^	1.77 ± 0.09	1.67 ± 0.10	2.01 ± 0.15	1.91 ± 0.13
Resting HR (bpm) *^a^*	62 ± 9	58 ± 11	60 ± 9	54 ± 10
Systolic BP (mmHg) *^b^*	115 ± 14	120 ± 13	128 ± 12	124 ± 12
Diastolic BP (mmHg)	72 ± 8	70 ± 9	73 ± 8	71 ± 9
Training duration (y)	11 ± 5	12 ± 5	12 ± 5	12 ± 5
Endurance METS p/w (METS) *^a^*	55 ± 17	147 ± 28	53 ± 19	159 ± 33
Sprint METS p/w (METS) *^a^*	122 ± 37	63 ± 12	117 ± 42	68 ± 14
Strength METS p/w (METS) *^a^*	30 ± 9	11 ± 2	29 ± 10	12 ± 3
Total METS p/w (METS) *^a^*	206 ± 62	221 ± 42	199 ± 71	240 ± 50

Notes. Data are presented mean ± SD. Abbreviations: BP, blood pressure; BSA, body surface area; HR, heart rate; METS, metabolic equivalents; p/w, per week. *^a^* Main effect of discipline (*p* < 0.05). *^b^* Main effect of sex (*p* < 0.05).

**Table 2:** Right ventricular structural and functional echocardiographic characteristics of elite cyclists.

	Female		Male	
	Sprint	Endurance	Sprint	Endurance
Structural parameters				
RVOT_plax_ (mm) *^a^*^,*b*^	27 ± 4	28 ± 3	30 ± 4	31 ± 4
RVOT_plax_ index (mm/m^2^)^(0.5)^ *^a^*	20 ± 3	22 ± 2	21 ± 3	22 ± 3
RVOT_1_ (mm) *^a^*^,*b*^	27 ± 3	29 ± 4	32 ± 4	33 ± 3
RVOT_1_ index (mm/m^2^)^(0.5)^ *^a^*^,*b*^	20 ± 2	22 ± 3	22 ± 3	24 ± 2
RVOT_2_ (mm) *^a^*^,*b*^	22 ± 2	24 ± 2	25 ± 3	27 ± 3
RVOT_2_ index (mm/m^2^)^(0.5)^ *^a^*^,*b*^	17 ± 2	18 ± 2	17 ± 2	19 ± 2
RVD_1_ (mm) *^a^*^,*b*^	37 ± 4	40 ± 4	42 ± 4	45 ± 5
RVD_1_ index (mm/m^2^)^(0.5)^ *^a^*^,*b*^	28 ± 3	31 ± 3	29 ± 3	32 ± 3
RVD_2_ (mm) *^b^*	27 ± 4	28 ± 4	31 ± 6	32 ± 4
RVD_2_ index (mm/m^2^)^(0.5)^ *^a^*^,*b*^	20 ± 3	22 ± 3	22 ± 4	23 ± 3
RVD_3_ (mm) *^a^*^,*b*^	78 ± 8	82 ± 7	85 ± 13	91 ± 10
RVD_3_ index (mm/m^2^)^(0.5)^ *^a^*	59 ± 5	63 ± 5	60 ± 9	66 ± 7
RVEDA (cm^2^) *^a^*^,*b*^	19 ± 4	22 ± 4	25 ± 4	27 ± 4
RVEDA index (cm^2^/m^2^) *^a^*^,*b*^	11 ± 2	13 ± 2	13 ± 2	14 ± 2
RVESA (cm^2^) *^a^*^,*b*^	11 ± 2	12 ± 2	14 ± 3	16 ± 3
RVESA index (cm^2^/m^2^) *^a^*^,*b*^	6 ± 1	7 ± 1	7 ± 1	8 ± 2
RV:LV ratio *^a^*^,*b*^	0.80 ± 0.1	0.88 ± 0.1	0.87 ± 0.1	0.91 ± 0.1
RVOT_1_:RVD_1_ ratio	0.76 ± 0.1	0.73 ± 0.1	0.76 ± 0.1	0.75 ± 0.1
EId	1.18 ± 0.1	1.19 ± 0.1	1.15 ± 0.1	1.19 ± 0.1
Eis *^b^*	0.96 ± 0.1	0.97 ± 0.1	1.0 ± 0.1	1.02 ± 0.1
Functional parameters				
RVFAC (%)	45 ± 7	45 ± 7	44 ± 8	43 ± 6
TAPSE (mm) *^b^*	22 ± 4	23 ± 4	23 ± 3	24 ± 4
RV S′ (cm/s) *^a^*^,*b*^	13 ± 2	14 ± 2	14 ± 2	14 ± 3
RV E′ (cm/s)	14 ± 2	14 ± 2	14 ± 3	13 ± 3
RV A′ (cm/s)	9 ± 2	9 ± 3	10 ± 3	10 ± 3
RV E′/A′ ratio	1.6 ± 0.5	1.6 ± 0.4	1.5 ± 0.5	1.5 ± 0.5
RVFWS (%) *^b^*	−26 ± 3	−25 ± 3	−24 ± 3	−23 ± 7
RVBS (%) *^a^*^,*b*^	−22 ± 5	−20 ± 4	−19 ± 5	−18 ± 4
RVMS (%)	−30 ± 4	−29 ± 5	−28 ± 6	−28 ± 5
RVAS (%) *^b^*	−26 ± 4	−27 ± 4	−24 ± 4	−25 ± 5
B-A gradient (%) *^a^*	−4 ± 7	−7 ± 5	−5 ± 6	−7 ± 6

*Notes.* Data are presented mean ± SD. Abbreviations: B-A, base-to-apex; EId, diastolic eccentricity index; Eis, systolic eccentricity index; FAC, fractional area change; LV, left ventricle; RV, right ventricle; RV A’, right ventricular late diastolic tissue velocity; RV E’, right ventricular early diastolic tissue velocity; RV S’, right ventricular systolic tissue velocity; RVAS, right ventricle apical strain; RVBS, right ventricle basal strain; RVD, right ventricle diameter; RVEDA, right ventricle end diastolic area; RVFWS, right ventricle free wall strain; RVMS, right ventricle mid strain; RVOT, right ventricular outflow tract; RVESA, right ventricle end systolic area; TAPSE, tricuspid annular systolic plane excursion. *^a^* Main effect of discipline (*p* < 0.05). *^b^* Main effect of sex (*p* < 0.05).

**Table 3:** Right atrial structural and functional echocardiographic characteristics of elite cyclists.

	Female		Male	
	Sprint	Endurance	Sprint	Endurance
RA area (cm^2^) *^a^*^,*b*^	14 ± 3	17 ± 4	17 ± 3	21 ± 4
RA area index (cm^2^/m^2^) *^a^*^,*b*^	8 ± 2	10 ± 2	9 ± 1	11 ± 2
RAESV (mL) *^a^*^,*b*^	23 ± 7	33 ± 11	57 ± 14	80 ± 23
RAESV index (mL/m^2^)^(1.5)^ *^a^*^,*b*^	17 ± 5	25 ± 8	20 ± 4	30 ± 9
RARV (mL) *^a^*^,*b*^	10 ± 4	14 ± 6	29 ± 9	37 ± 15
RARV index (mL/m^2^)^(1.5)^ *^a^*^,*b*^	7 ± 3	11 ± 5	10 ± 3	14 ± 5
RACV (mL) *^b^*	27 ± 9	31 ± 8	55 ± 17	60 ± 18
RACV index (mL/m^2^)^(1.5)^ *^a^*	21 ± 7	24 ± 7	19 ± 6	23 ± 7
RABV (mL) *^a^*^,*b*^	4 ± 2	6 ± 3	12 ± 5	15 ± 6
RABV index (mL/m^2^)^(1.5)^ *^a^*^,*b*^	3 ± 2	4 ± 2	4 ± 2	6 ± 2
IVC diameter (mm) *^a^*^,*b*^	19 ± 4	22 ± 4	21 ± 4	25 ± 4
RARS (%)	34 ± 9	37 ± 6	34 ± 8	34 ± 8
RACS (%)	24 ± 7	26 ± 5	25 ± 6	25 ± 1
RABS (%)	11 ± 5	12 ± 3	11 ± 4	11 ± 4

*Notes.* Data are presented as mean ± SD. Abbreviations: IVC, inferior vena cava; RA, right atrium; RABS, right atrial booster strain; RABV, right atrial booster volume; RACS, right atrial conduit strain; RACV, right atrial conduit volume; RAESV, right atrial end systolic volume; RARS, right atrial reservoir strain; RARV, right atrial reservoir volume. *^a^* Main effect of discipline (*p* < 0.05). *^b^* Main effect of sex (*p* < 0.05).

## Data Availability

Data is available upon request.

## Abbreviations

The following abbreviations are used in this manuscript:

2D	Two-dimensional
A4C	Apical four chamber
B-A	Base-to-apex
BSA	Body surface area
BSE	British Society of Echocardiography
CO	Cardiac output
CoV	Coefficient of variation
DICOM	Digital imaging and communications in medicine
ECG	Electrocardiogram
EId	Eccentricity index (diastole)
EIs	Eccentricity index (systole)
FE	Female endurance
FS	Female sprint
HR	Heart rate
ICC	Intraclass correlation
IVC	Inferior vena cava
LV	Left ventricle
ME	Male endurance
MET	Metabolic equivalent
MS	Male sprint
PAP	Pulmonary artery pressure
RA	Right atrium
RABS	Right atrial booster strain
RABV	Right atrial booster volume
RACS	Right atrial conduit strain
RACV	Right atrial conduit volume
RAEDV	Right atrial end diastolic volume
RAESV	Right atrial end systolic volume
RARS	Right atrial reservoir strain
RARV	Right atrial reservoir volume
RV	Right ventricle
RV A’	Right ventricular late diastolic tissue velocity
RV E’	Right ventricular early diastolic tissue velocity
RV S’	Right ventricular systolic tissue velocity
RVAS	Right ventricular apical strain
RVBS	Right ventricular basal strain
RVD_1_	Right ventricle basal-level diameter
RVD_2_	Right ventricle mid-level diameter
RVD_3_	Right ventricle length diameter
RVEDA	Right ventricular end diastolic area
RVESA	Right ventricular end systolic area
RVFAC	Right ventricular fractional area change
RVFW	Right ventricular free wall
RVMS	Right ventricular mid strain
RVOT	Right ventricular outflow tract
RVOT_1_	Right ventricular outflow tract anterior wall to free wall diameter
RVOT_2_	Right ventricular outflow tract proximal to pulmonary valve diameter
RVOT_plax_	Right ventricular outflow tract interventricular septum to free wall diameter
SBP	Systolic blood pressure
SD	Standard deviation
STE	Speckle-tracking echocardiography
TAPSE	Tricuspid annular plane systolic excursion
VO_2_max	Maximal oxygen consumption
WADA	World Anti Doping Agency

## References

[1] Pelliccia A., Caselli S., Sharma S., Basso C., Bax J.J., Corrado D., D’Andrea A., D’Ascenzi F., Di Paolo F.M., Edvardsen T. (2018). European Association of Preventive Cardiology (EAPC) and European Association of Cardiovascular Imaging (EACVI) joint position statement: Recommendations for the indication and interpretation of cardiovascular imaging in the evaluation of the athlete’s heart. Eur. Heart J..

[2] Gülan U., Rossi V.A., Gotschy A., Saguner A.M., Manka R., Brunckhorst C.B., Duru F., Schmied C.M., Niederseer D. (2022). A comparative study on the analysis of hemodynamics in the athlete’s heart. Sci. Rep..

[3] Brown B., Somauroo J., Green D.J., Wilson M., Drezner J., George K., Oxborough D. (2017). The Complex Phenotype of the Athlete’s Heart: Implications for Preparticipation Screening. Exerc. Sport. Sci. Rev..

[4] Levine B.D., Baggish A.L., Kovacs R.J., Link M.S., Maron M.S., Mitchell J.H. (2015). Eligibility and Disqualification Recommendations for Competitive Athletes With Cardiovascular Abnormalities: Task Force 1: Classification of Sports: Dynamic, Static, and Impact: A Scientific Statement From the American Heart Association and American College of Cardiology. J. Am. Coll. Cardiol..

[5] D’Ascenzi F., Biella F., Lemme E., Maestrini V., Di Giacinto B., Pelliccia A. (2020). Female Athlete’s Heart: Sex Effects on Electrical and Structural Remodeling. Circ. Cardiovasc. Imaging.

[6] D’Andrea A., Riegler L., Golia E., Cocchia R., Scarafile R., Salerno G., Pezzullo E., Nunziata L., Citro R., Cuomo S. (2013). Range of right heart measurements in top-level athletes: The training impact. Int. J. Cardiol..

[7] Kovacs R., Baggish A.L. (2016). Cardiovascular adaptation in athletes. Trends Cardiovasc. Med..

[8] Brown B., Millar L., Somauroo J., George K., Sharma S., La Gerche A., Forsythe L., Oxborough D. (2020). Left ventricular remodeling in elite and sub-elite road cyclists. Scand. J. Med. Sci. Sports.

[9] Abergel E., Chatellier G., Hagege A.A., Oblak A., Linhart A., Ducardonnet A., Menard J. (2004). Serial left ventricular adaptations in world-class professional cyclists: Implications for disease screening and follow-up. J. Am. Coll. Cardiol..

[10] D’Ascenzi F., Pisicchio C., Caselli S., Di Paolo F.M., Spataro A., Pelliccia A. (2017). RV Remodeling in Olympic Athletes. JACC Cardiovasc. Imaging.

[11] Oxborough D., Sharma S., Shave R., Whyte G., Birch K., Artis N., Batterham A.M., George K. (2012). The right ventricle of the endurance athlete: The relationship between morphology and deformation. J. Am. Soc. Echocardiogr..

[12] Heidbüchel H., La Gerche A. (2012). The right heart in athletes. Evidence for exercise-induced arrhythmogenic right ventricular cardiomyopathy. Herzschrittmacherther. Elektrophysiol..

[13] Oxborough D., Shave R., Warburton D., Williams K., Oxborough A., Charlesworth S., Foulds H., Hoffman M.D., Birch K., George K. (2011). Dilatation and dysfunction of the right ventricle immediately after ultraendurance exercise: Exploratory insights from conventional two-dimensional and speckle tracking echocardiography. Circ. Cardiovasc. Imaging.

[14] Flanagan H., Cooper R., George K.P., Augustine D.X., Malhotra A., Paton M.F., Robinson S., Oxborough D. (2023). The athlete’s heart: Insights from echocardiography. Echo Res. Pract..

[15] La Gerche A., Burns A.T., Taylor A.J., Macisaac A.I., Heidbüchel H., Prior D.L. (2012). Maximal oxygen consumption is best predicted by measures of cardiac size rather than function in healthy adults. Eur. J. Appl. Physiol..

[16] Lazic J.S., Tadic M., Antic M., Radovanovic D., Nesic D., Rakocevic R., Mazic S. (2019). The relationship between right heart and aerobic capacity in large cohort of young elite athletes. Int. J. Cardiovasc. Imaging.

[17] Wundersitz D.W.T., Gordon B.A., Lavie C.J., Nadurata V., Kingsley M.I.C. (2020). Impact of endurance exercise on the heart of cyclists: A systematic review and meta-analysis. Prog. Cardiovasc. Dis..

[18] Pluim B.M., Zwinderman A.H., van der Laarse A., van der Wall E.E. (2000). The athlete’s heart. A meta-analysis of cardiac structure and function. Circulation.

[19] Conti V., Migliorini F., Pilone M., Barriopedro M.I., Ramos-Álvarez J.J., Montero F.J.C., Maffulli N. (2021). Right heart exercise-training-adaptation and remodelling in endurance athletes. Sci. Rep..

[20] Gerche A.L., Wasfy M.M., Brosnan M.J., Claessen G., Fatkin D., Heidbuchel H., Baggish A.L., Kovacic J.C. (2022). The Athlete’s Heart—Challenges and Controversies. JACC.

[21] D’Ascenzi F., Cameli M., Padeletti M., Lisi M., Zacà V., Natali B., Malandrino A., Alvino F., Morelli M., Vassallo G.M. (2013). Characterization of right atrial function and dimension in top-level athletes: A speckle tracking study. Int. J. Cardiovasc. Imaging.

[22] Sanz J., Sánchez-Quintana D., Bossone E., Bogaard H.J., Naeije R. (2019). Anatomy, Function, and Dysfunction of the Right Ventricle: JACC State-of-the-Art Review. J. Am. Coll. Cardiol..

[23] Sanz-de la Garza M., Giraldeau G., Marin J., Grazioli G., Esteve M., Gabrielli L., Brambila C., Sanchis L., Bijnens B., Sitges M. (2017). Influence of gender on right ventricle adaptation to endurance exercise: An ultrasound two-dimensional speckle-tracking stress study. Eur. J. Appl. Physiol..

[24] Giraldeau G., Kobayashi Y., Finocchiaro G., Wheeler M., Perez M., Kuznetsova T., Lord R., George K.P., Oxborough D., Schnittger I. (2015). Gender differences in ventricular remodeling and function in college athletes, insights from lean body mass scaling and deformation imaging. Am. J. Cardiol..

[25] D’Ascenzi F., Pelliccia A., Natali B.M., Zacà V., Cameli M., Alvino F., Malandrino A., Palmitesta P., Zorzi A., Corrado D. (2014). Morphological and functional adaptation of left and right atria induced by training in highly trained female athletes. Circ. Cardiovasc. Imaging.

[26] Herrmann S.D., Willis E.A., Ainsworth B.E., Barreira T.V., Hastert M., Kracht C.L., Schuna J.M., Cai Z., Quan M., Tudor-Locke C. (2024). 2024 Adult Compendium of Physical Activities: A third update of the energy costs of human activities. J. Sport Health Sci..

[27] Mosteller R.D. (1987). Simplified calculation of body-surface area. N. Engl. J. Med..

[28] Oxborough D., George K., Cooper R., Bhatia R., Ramcharan T., Zaidi A., Gati S., Prakash K., Rakhit D., Robinson S. (2025). Echocardiography in the cardiac assessment of young athletes: A 2025 guideline from the British Society of Echocardiography (endorsed by Cardiac Risk in the Young). Echo Res. Pract..

[29] Oxborough D., Augustine D., Gati S., George K., Harkness A., Mathew T., Papadakis M., Ring L., Robinson S., Sandoval J. (2018). A guideline update for the practice of echocardiography in the cardiac screening of sports participants: A joint policy statement from the British Society of Echocardiography and Cardiac Risk in the Young. Echo Res. Pract..

[30] Robinson S., Rana B., Oxborough D., Steeds R., Monaghan M., Stout M., Pearce K., Harkness A., Ring L., Paton M. (2020). A practical guideline for performing a comprehensive transthoracic echocardiogram in adults: The British Society of Echocardiography minimum dataset. Echo Res. Pract..

[31] Dewey F.E., Rosenthal D., Murphy D.J., Froelicher V.F., Ashley E.A. (2008). Does size matter? Clinical applications of scaling cardiac size and function for body size. Circulation.

[32] Buckberg G., Hoffman J.I. (2014). Right ventricular architecture responsible for mechanical performance: Unifying role of ventricular septum. J. Thorac. Cardiovasc. Surg..

[33] Oxborough D., George K., Birch K.M. (2012). Intraobserver reliability of two-dimensional ultrasound derived strain imaging in the assessment of the left ventricle, right ventricle, and left atrium of healthy human hearts. Echocardiography.

[34] Lord R.N., George K., Jones H., Somauroo J., Oxborough D. (2014). Reproducibility and feasibility of right ventricular strain and strain rate (SR) as determined by myocardial speckle tracking during high-intensity upright exercise: A comparison with tissue Doppler-derived strain and SR in healthy human hearts. Echo Res. Pract..

[35] Cohen J. (2013). Statistical Power Analysis for the Behavioral Sciences.

[36] Arbab-Zadeh A., Perhonen M., Howden E., Peshock R.M., Zhang R., Adams-Huet B., Haykowsky M.J., Levine B.D. (2014). Cardiac remodeling in response to 1 year of intensive endurance training. Circulation.

[37] Dawkins T.G., Curry B.A., Wright S.P., Meah V.L., Yousef Z., Eves N.D., Shave R.E., Stembridge M. (2021). Right Ventricular Function and Region-Specific Adaptation in Athletes Engaged in High-Dynamic Sports: A Meta-Analysis. Circ. Cardiovasc. Imaging.

[38] Lakatos B.K., Kiss O., Tokodi M., Tősér Z., Sydó N., Merkely G., Babity M., Szilágyi M., Komócsin Z., Bognár C. (2018). Exercise-induced shift in right ventricular contraction pattern: Novel marker of athlete’s heart?. Am. J. Physiol. Heart Circ. Physiol..

[39] Kannan A., Poongkunran C., Jayaraj M., Janardhanan R. (2014). Role of strain imaging in right heart disease: A comprehensive review. J. Clin. Med. Res..

[40] Ho S.Y., Nihoyannopoulos P. (2006). Anatomy, echocardiography, and normal right ventricular dimensions. Heart.

[41] La Gerche A., Heidbüchel H., Burns A.T., Mooney D.J., Taylor A.J., Pfluger H.B., Inder W.J., Macisaac A.I., Prior D.L. (2011). Disproportionate exercise load and remodeling of the athlete’s right ventricle. Med. Sci. Sports Exerc..

[42] Stefani L., Pedrizzetti G., De Luca A., Mercuri R., Innocenti G., Galanti G. (2009). Real-time evaluation of longitudinal peak systolic strain (speckle tracking measurement) in left and right ventricles of athletes. Cardiovasc. Ultrasound.

[43] La Gerche A., Rakhit D.J., Claessen G. (2017). Exercise and the right ventricle: A potential Achilles’ heel. Cardiovasc. Res..

[44] Sanz-de la Garza M., Carro A., Caselli S. (2020). How to interpret right ventricular remodeling in athletes. Clin. Cardiol..

[45] Reeves J.T., Groves B.M., Cymerman A., Sutton J.R., Wagner P.D., Turkevich D., Houston C.S. (1990). Operation Everest II: Cardiac filling pressures during cycle exercise at sea level. Respir. Physiol..

[46] McGill H.C., Anselmo V.C., Buchanan J.M., Sheridan P.J. (1980). The heart is a target organ for androgen. Science.

[47] Huang C.K., Lee S.O., Chang E., Pang H., Chang C. (2016). Androgen receptor (AR) in cardiovascular diseases. J. Endocrinol..

[48] Parker T.A., Ivy D.D., Galan H.L., Grover T.R., Kinsella J.P., Abman S.H. (2000). Estradiol improves pulmonary hemodynamics and vascular remodeling in perinatal pulmonary hypertension. Am. J. Physiol. Lung Cell Mol. Physiol..

[49] Tofovic S.P. (2010). Estrogens and development of pulmonary hypertension: Interaction of estradiol metabolism and pulmonary vascular disease. J. Cardiovasc. Pharmacol..

[50] Beaumont A., Grace F., Richards J., Hough J., Oxborough D., Sculthorpe N. (2017). Left Ventricular Speckle Tracking-Derived Cardiac Strain and Cardiac Twist Mechanics in Athletes: A Systematic Review and Meta-Analysis of Controlled Studies. Sports Med..

[51] Teske A.J., Prakken N.H., De Boeck B.W., Velthuis B.K., Martens E.P., Doevendans P.A., Cramer M.J. (2009). Echocardiographic tissue deformation imaging of right ventricular systolic function in endurance athletes. Eur. Heart J..

[52] La Gerche A., Burns A.T., Mooney D.J., Inder W.J., Taylor A.J., Bogaert J., MacIsaac A.I., Heidbüchel H., Prior D.L. (2011). Exercise-induced right ventricular dysfunction and structural remodelling in endurance athletes. Eur. Heart J..

[53] Forsythe L., Somauroo J., George K., Papadakis M., Brown B., Qasem M., Oxborough D. (2019). The right heart of the elite senior rugby football league athlete. Echocardiography.

[54] Utomi V., Oxborough D., Ashley E., Lord R., Fletcher S., Stembridge M., Shave R., Hoffman M.D., Whyte G., Somauroo J. (2015). The impact of chronic endurance and resistance training upon the right ventricular phenotype in male athletes. Eur. J. Appl. Physiol..

[55] La Gerche A., Burns A.T., D’Hooge J., Macisaac A.I., Heidbüchel H., Prior D.L. (2012). Exercise strain rate imaging demonstrates normal right ventricular contractile reserve and clarifies ambiguous resting measures in endurance athletes. J. Am. Soc. Echocardiogr..

[56] Gabrielli L., Bijnens B.H., Butakoff C., Duchateau N., Montserrat S., Merino B., Gutierrez J., Pare C., Mont L., Brugada J. (2014). Atrial functional and geometrical remodeling in highly trained male athletes: For better or worse?. Eur. J. Appl. Physiol..

[57] Rudski L.G., Lai W.W., Afilalo J., Hua L., Handschumacher M.D., Chandrasekaran K., Solomon S.D., Louie E.K., Schiller N.B. (2010). Guidelines for the echocardiographic assessment of the right heart in adults: A report from the American Society of Echocardiography endorsed by the European Association of Echocardiography, a registered branch of the European Society of Cardiology, and the Canadian Society of Echocardiography. J. Am. Soc. Echocardiogr..

[58] Popović Z.B., Sun J.P., Yamada H., Drinko J., Mauer K., Greenberg N.L., Cheng Y., Moravec C.S., Penn M.S., Mazgalev T.N. (2005). Differences in left ventricular long-axis function from mice to humans follow allometric scaling to ventricular size. J. Physiol..

[59] Batterham A., Shave R., Oxborough D., Whyte G., George K. (2008). Longitudinal plane colour tissue-Doppler myocardial velocities and their association with left ventricular length, volume, and mass in humans. Eur. J. Echocardiogr..

[60] Ujka K., Bastiani L., D’Angelo G., Catuzzo B., Tonacci A., Mrakic-Sposta S., Vezzoli A., Giardini G., Pratali L. (2017). Enhanced Right-Chamber Remodeling in Endurance Ultra-Trail Athletes Compared to Marathon Runners Detected by Standard and Speckle-Tracking Echocardiography. Front. Physiol..

[61] Pagourelias E.D., Kouidi E., Efthimiadis G.K., Deligiannis A., Geleris P., Vassilikos V. (2013). Right atrial and ventricular adaptations to training in male Caucasian athletes: An echocardiographic study. J. Am. Soc. Echocardiogr..

[62] McClean G., George K., Lord R., Utomi V., Jones N., Somauroo J., Fletcher S., Oxborough D. (2015). Chronic adaptation of atrial structure and function in elite male athletes. Eur. Heart J. Cardiovasc. Imaging.

[63] Zaidi A., Ghani S., Sharma R., Oxborough D., Panoulas V.F., Sheikh N., Gati S., Papadakis M., Sharma S. (2013). Physiological right ventricular adaptation in elite athletes of African and Afro-Caribbean origin. Circulation.

[64] Santarpino G., Taverna G., Calabrese V., Coviello F., Trimarchi G., Trio O., Fiore C., Andò G., Nasso G., Speziale G. (2025). Exploring Imaging Depth: A Pilot Study About 2D vs. 4D Echocardiography for Tricuspid Valve Evaluation. Rev. Cardiovasc. Med..

